# Treatment of Prostatic Hypertrophy by Injection of Animal Blood

**Published:** 1909-07

**Authors:** 


### Treatment of Prostatic Hypertrophy by Injection of Animal Blood.

According to Bier's investigation the injection into human tissues of blood taken from animals causes inflammation followed by contraction. This observation has led Dr. Jüngling to ascertain the possibility of reducing prostatic hypertrophy by means of injections of defibrinated hog or lamb blood. The blood was injected into the prostate with a hypodermic syringe through the perineum under control of the finger inserted into the rectum. About 2½ c. cm. was injected into each lobe and the same amount into the periprostatic tissue. This method was tried in twenty-one cases and proved to be perfectly free from risk. In several cases after its use the urinary stream became more forcible and the frequency of urination was reduced, while in cases attended with cystitis this improved without resort to local treatment. Owing to the difficulty of determining slight changes in the size of the enlarged prostate, it could not be demonstrated whether the injections had any direct influence in this respect.—*International Journal of Surgery.*

---

It is easy enough to be pleasant
When life goes on like a song;
But the man worth while is the man who can smile
When the telephone rings and he answers it
And says "Hello," and the operator says
"What number?" and he says, "The bell rang,"
And then she says, "No, it didn't."
—*New York Mail.*

---

The surgeon should keep closely in touch with cases of acute retropharyngeal abscess, as serious edema of the glottis may develop and require tracheotomy for its relief.—*American Journal of Surgery.*

FAT SHOULD BE INCREASED AS WE GROW OLDER.—As we grow older, become more energetic in mind and body, and as the increased surface of our body requires more fuel to maintain the heat of our body and for increased energy for movement, to be in the highest state of health and to possess the greatest amount of energy, we must assimilate that greatest of all dynamic heat and force-giving food—fat. Some have talked wisely about establishing fat equilibrium. But I maintain that there should be more fat eaten than that which is just required to maintain heat and energy at its lowest. We should have sufficient to run on six cylinders instead of one, and we should possess a plump amount of adipose tissue to be drawn upon in case of need, a reserve tissue or bank account, as it were. Then we possess that abundant healthy life force, we have described as the acme of healthy temperament or constitution.—*T. B. Keyes, in Pac. Med.*

